# Sub-atomic resolution X-ray crystallography and neutron crystallography: promise, challenges and potential

**DOI:** 10.1107/S2052252515011239

**Published:** 2015-06-30

**Authors:** Matthew P. Blakeley, Samar S. Hasnain, Svetlana V. Antonyuk

**Affiliations:** a Large-Scale Structures Group, Institut Laue-Langevin, 71 Avenue des Martyrs, Grenoble 38000, France; b Molecular Biophysics Group, Institute of Integrative Biology, Faculty of Health and Life Sciences, University of Liverpool, Liverpool L69 7ZX, UK

**Keywords:** neutron, X-ray, hydrogen, proton, protonation states, radiation damage, redox biology, proton coupling, electron transfer, X-ray laser, XFEL

## Abstract

The International Year of Crystallography saw the number of macromolecular structures deposited in the Protein Data Bank cross the 100000 mark, with more than 90000 of these provided by X-ray crystallography. The number of X-ray structures determined to sub-atomic resolution (*i.e.* ≤1 Å) has passed 600 and this is likely to continue to grow rapidly with diffraction-limited synchrotron radiation sources such as MAX-IV (Sweden) and Sirius (Brazil) under construction. A dozen X-ray structures have been deposited to ultra-high resolution (*i.e.* ≤0.7 Å), for which precise electron density can be exploited to obtain charge density and provide information on the bonding character of catalytic or electron transfer sites. Although the development of neutron macromolecular crystallography over the years has been far less pronounced, and its application much less widespread, the availability of new and improved instrumentation, combined with dedicated deuteration facilities, are beginning to transform the field. Of the 83 macromolecular structures deposited with neutron diffraction data, more than half (49/83, 59%) were released since 2010. Sub-mm^3^ crystals are now regularly being used for data collection, structures have been determined to atomic resolution for a few small proteins, and much larger unit-cell systems (cell edges >100 Å) are being successfully studied. While some details relating to H-atom positions are tractable with X-ray crystallography at sub-atomic resolution, the mobility of certain H atoms precludes them from being located. In addition, highly polarized H atoms and protons (H^+^) remain invisible with X-rays. Moreover, the majority of X-ray structures are determined from cryo-cooled crystals at 100 K, and, although radiation damage can be strongly controlled, especially since the advent of shutterless fast detectors, and by using limited doses and crystal translation at micro-focus beams, radiation damage can still take place. Neutron crystallography therefore remains the only approach where diffraction data can be collected at room temperature without radiation damage issues and the only approach to locate mobile or highly polarized H atoms and protons. Here a review of the current status of sub-atomic X-ray and neutron macromolecular crystallography is given and future prospects for combined approaches are outlined. New results from two metalloproteins, copper nitrite reductase and cytochrome *c*′, are also included, which illustrate the type of information that can be obtained from sub-atomic-resolution (∼0.8 Å) X-ray structures, while also highlighting the need for complementary neutron studies that can provide details of H atoms not provided by X-ray crystallography.

## Abstract 2

Neutron crystallography and sub-atomic X-ray crystallography complement each other in defining hydrogen positions in macromolecules. Significant advances have been made but much effort is still required if neutron crystallography is to become a mainstream activity.

## 1. Introduction   

The tremendous success of X-ray crystallography over the last 30 years has largely been due to the availability of highly intense synchrotron radiation (SR) facilities. More recently, the drive to ever-increasing brightness of X-ray sources has led to the establishment of X-ray free-electron laser (FEL) facilities in the USA and Japan, with new facilities under construction in Europe, South East Asia and elsewhere (Weckert, 2015 ▸). X-ray FELs provide huge gains in intensity, allowing molecular structures to be obtained from crystals of a few nanometres (Chapman *et al.*, 2011 ▸), and, ultimately, the possibility of visualizing macromolecular structures and complexes at high resolution without the need for crystals at all (Stern *et al.*, 2014 ▸). X-ray diffraction data extending to sub-atomic resolution (*i.e.* ≤1 Å) leads to extremely precise electron density maps that allow the positions of a significant proportion of H atoms to be determined. Knowledge of the H-atom positions provides detailed information on protonation states (*e.g.* for amino-acid side-chains, bound drugs/inhibitors *etc*.), water structure and hydrogen bonding, which can be critical towards understanding macromolecular function (Kosinka Eriksson *et al.*, 2013 ▸; Matsuoka *et al.*, 2015 ▸; Ogata *et al.*, 2015 ▸). Moreover, charge density information can be obtained, as illustrated by the studies of crambin (Jelsch *et al.*, 2000 ▸), aldose reductase (Guillot *et al.*, 2008 ▸) and cholesterol oxidase (Zarychta *et al.*, 2015 ▸). Neutron crystallography provides an alternative approach to sub-atomic X-ray crystallography for the location of H atoms and is the only approach for the location of highly polarized H atoms and protons (H^+^) since these are invisible with X-rays. As the coherent neutron scattering length of H and its isotope deuterium (D) are of similar magnitude to the other common elements of a macromolecule (Table 1 ▸), their positions can be located at much lower resolutions than required with X-rays. Furthermore, H atoms observed in electron density maps at sub-atomic resolution are generally those with low thermal motion while the more mobile H atoms, often the most biologically interesting, tend to remain invisible, as their already weak scattering signal is further smeared out. Neutron crystallography has been shown to provide the positions of H atoms that are difficult to observe in sub-atomic electron density maps, despite the much lower resolutions of the neutron diffraction data sets (Gardberg *et al.*, 2010 ▸).

A further advantage of using neutrons is that they do not cause any observable radiation damage to the crystal, such that data can be collected at room temperature, avoiding any potential cryo-cooling effects (Keedy *et al.*, 2014 ▸) and resulting in the determination of damage-free structures. This is of particular significance since, despite all the advances in X-ray detectors and data collection protocols, radiation damage can still occur at 100 K (Liebschner *et al.*, 2013 ▸; Kekilli *et al.*, 2014 ▸). This is particularly evident for redox centres in metalloproteins (Kekilli *et al.*, 2014 ▸; Suga *et al.*, 2015 ▸) and carboxylic-acid-containing residues, Asp and Glu, since X-rays cause CO_2_ elimination, precluding determination of Asp and Glu protonation states (Gerstel *et al.*, 2015 ▸). We note that, despite the large number of X-ray structures in the Protein Data Bank (PDB), there is still a scarcity of genuine ‘damage-free’ structures of redox systems at ambient temperatures.

**Figure 1:**
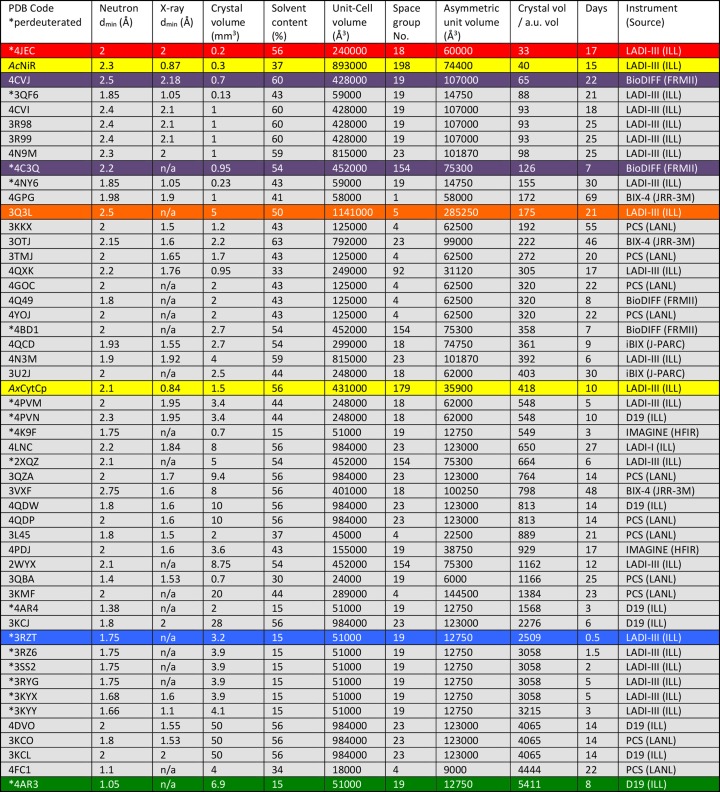
Neutron and joint X-ray/neutron structures of macromolecules deposited in the PDB since 2010, including data collection details and crystallographic parameters for each. The structures are ordered in terms of the ratio of the crystal volume to the asymmetric unit volume, from lowest to highest. Those with the lowest ratios can be considered the most challenging. Highlighted in red is the study of HIV-1 protease with the antiretroviral drug amprenavir bound (Weber *et al.*, 2013 ▸) that has the lowest ratio of the crystal volume to the asymmetric unit volume. Highlighted in orange is the study of inorganic pyrophosphatase from *Thermococcus thioreducens* (I-PPase; Hughes *et al.*, 2012 ▸) that currently is the largest unit cell and asymmetric unit volume to be studied. Highlighted in blue is a study of rubredoxin from *Pyrococcus furiosus* (RdPf; Munshi *et al.*, 2012 ▸) that is the fastest data collection to date at 14 h. Highlighted in green is another study of RdPf (Cuypers *et al.*, 2013 ▸) which is currently the highest resolution study at 1.05 Å. Highlighted in purple are neutron cryo-crystallography studies performed at 100 K for cytochrome *c* peroxidase (MW ∼34 kDa) (Casadei *et al.*, 2014 ▸) and β-lactamase (MW ∼28 kDa) (Coates *et al.*, 2014 ▸), and highlighted in yellow are the studies of Cu nitrite reductase (MW ∼37 kDa) from *Achromobacter cycloclastes* (*Ac*NiR) and cytochrome *c*′ (MW ∼14 kDa) from *Alcaligenes xylosoxidans* (*Ax*CytCp) presented here.

The application of neutron crystallography for the location of H atoms in macromolecules has historically been restricted to the study of small unit-cell systems (cell edges <30 Å) for which large crystals of several mm^3^ could be grown. Even then, long data collection times of several months were required due to the lack of optimized instrumentation (Schoenborn, 2010 ▸). This is no longer the case, with neutron crystallography expanding beyond its traditional boundaries, to address larger and more complex problems, with smaller samples and shorter data collection times. The origin of this transformation can be found in a number of advances including the development of quasi-Laue (Blakeley *et al.*, 2010 ▸; Meilleur *et al.*, 2013 ▸) and monochromatic (Kurihara *et al.*, 2004 ▸; http://www.mlz-garching.de/biodiff) diffractometers with cylindrical image-plate detectors at nuclear reactor neutron sources, time-of-flight (TOF) Laue diffractometers at spallation neutron sources (Langan *et al.*, 2004 ▸; Coates *et al.*, 2010 ▸; Kusaka *et al.*, 2013 ▸), centralized facilities for sample deuteration and new computational tools for structural refinement (Afonine *et al.*, 2010 ▸; Gruene *et al.*, 2014 ▸). Of the 83 neutron structures in the PDB, more than half (49/83) were deposited since 2010 (Fig. 1 ▸). Many of these illustrate the complementarity of combining X-ray and neutron crystallography in order to locate important H-atom positions to improve our understanding of macromolecular structure and function.

## 2. H/D exchange and perdeuteration   

In neutron macromolecular crystallography, the replacement of H by D is advantageous for two main reasons. Firstly, H has an anomalously large incoherent scattering cross section, while that of D is ∼40 times lower (Table 1 ▸). Since H atoms constitute around half of the atoms in a macromolecular crystal, their incoherent scattering signal contributes significantly to a high scattered background; H/D isotopic replacement therefore enhances the signal-to-noise ratio of the diffraction data by lowering the incoherent background, thereby extending the resolution limit. Secondly, as the coherent scattering length of D is positive and approximately twice that of H, D atoms are more readily located in neutron maps than H atoms.

Neutron studies have been most commonly performed (64/83, 77%) using D-exchanged single crystals (Tomanicek *et al.*, 2010 ▸; Kovalevsky *et al.*, 2010 ▸, 2012 ▸; Fisher *et al.*, 2012 ▸; Yokoyama *et al.*, 2013 ▸; Casadei *et al.*, 2014 ▸; Huang *et al.*, 2014 ▸; Langan *et al.*, 2014 ▸; Oksanen *et al.*, 2014 ▸; Wan *et al.*, 2014 ▸; Unno *et al.*, 2015 ▸; Michalczyk *et al.*, 2015 ▸). H/D exchange can be achieved by vapor exchange or by soaking in D_2_O solutions and allows exchange of solvent-accessible H atoms attached to oxygen or nitrogen, but not those H atoms attached to carbon. Neutron data collected from D-exchanged crystals allows D atoms attached to O or N atoms to be readily visualized at 2.5 Å resolution. In order to readily locate H atoms, data must extend to ∼1.5 Å resolution (Chen *et al.*, 2012 ▸) since at lower resolutions cancellation effects (between positive and negative neutron scatterers) limit visualization of H atoms attached to C atoms (*e.g.* CH_2_, CH_3_ groups). Becoming more prevalent are studies performed using perdeuterated samples (19/83 structures overall, 23%; 17/49 structures since 2010, 35%), produced *via* bacterial expression on deuterated media (Petit-Haertlein *et al.*, 2009 ▸). As perdeuteration provides complete deuteration (*i.e.* all H replaced by D), neutron diffraction data from perdeuterated crystals have vastly improved signal-to-noise ratios, allowing shorter data collection times (Munshi *et al.*, 2012 ▸) and/or providing data to higher resolution (Cuypers *et al.*, 2013 ▸). Since the historical bottleneck for successful neutron crystallographic studies has been the need for sufficiently large crystal volumes, perhaps most importantly perdeuteration permits data collection from much smaller crystal volumes (*cf.* D-exchanged samples) (Howard *et al.*, 2011 ▸; Weber *et al.*, 2013 ▸), making larger unit-cell systems more accessible to study. Furthermore, neutron map cancellation effects are avoided, allowing all D atoms to be readily visualized (at 2.5 Å resolution), including those attached to carbon atoms (*e.g.* CD_2_, CD_3_ groups) (Fisher *et al.*, 2014 ▸). Neutron facilities throughout the world have now developed dedicated laboratories for deuteration, such as the Deuteration Laboratory at the Institut Laue-Langevin (ILL) and the Bio-Deuteration Laboratory at Oak Ridge National Laboratory (ORNL).

## 3. Neutron instrumentation at reactor neutron sources   

In recent years, great progress has been made in developing new and improved instrumentation for neutron macromolecular crystallography. At reactor neutron sources, the use of cylindrical neutron-sensitive image-plate (NIP) detectors that completely surround the sample and provide large coverage of reciprocal space (>2π steradian) have been incorporated into the design of instrumentation, such as the LADI-III diffractometer (Blakeley *et al.*, 2010 ▸) at the ILL high-flux reactor, the BioDIFF diffractometer (http://www.mlz-garching.de/biodiff) at the Forschungsreaktor Munchen II research reactor (FRM II), the BIX-3 and BIX-4 diffractometers (Tanaka *et al.*, 2002 ▸; Kurihara *et al.*, 2004 ▸) at the JRR-3M reactor at the Japan Atomic Energy Agency (JAEA) and the IMAGINE diffractometer (Meilleur *et al.*, 2013 ▸) at the High Flux Isotope Reactor (HFIR) at ORNL. In order to reduce the volumes of the crystals required, the LADI-III and IMAGINE diffractometers utilize quasi-Laue methods for data collection, in which a narrow-wavelength band pass (*e.g.* δλ/λ = 30%) is extracted from the original broadband spectrum of wavelengths (white beam). Quasi-Laue methods provide large gains in flux relative to monochromatic methods, while reducing background scattering and reflection overlap compared with the use of the full white beam. In 2012, the LADI-III diffractometer was relocated to a new guide (H143) closer to the ILL reactor. The new end-position provides an improved band-pass profile (due to the lack of instruments upstream) and most importantly a four-fold increase in flux at the sample position (*cf.* previous H142 position). Owing to these recent improvements, LADI-III is further extending the limits of the field. Currently the 2.0 Å resolution structure (PDB code: 4JEC) of perdeuterated HIV-1 protease (MW ∼21 kDa) with amprenavir bound (Weber *et al.*, 2013 ▸) has the lowest ratio (33) for the crystal volume used for data collection (0.2 mm^3^) to the asymmetric unit volume of the macromolecule studied (60000 Å^3^). Moreover, the 2.5 Å resolution structure (PDB code: 3Q3L) of inorganic pyrophosphatase (I-PPase, MW ∼125 kDa) has the largest asymmetric unit volume (*a* = 106.1 Å, *b* = 95.5 Å, *c* = 113.7 Å, β = 98.1°/*C*2) deposited thus far (Hughes *et al.*, 2012 ▸). This structure was determined using data from a 5 mm^3^ D-exchanged crystal of I-PPase; however, more recently data have been collected to ∼2.5 Å resolution using the LADI-III diffractometer but from a dramatically smaller (0.32 mm^3^) perdeuterated crystal of I-PPase, illustrating the huge benefits of perdeuteration (Ng/Garcia-Ruiz, unpublished results). The 1.05 Å resolution neutron structure (PDB code: 4AR3) of the oxidized form of perdeuterated rubredoxin from *Pyrococcus furiosus* (RdPf, MW ∼6 kDa) is to the highest resolution of any of the structures deposited thus far (Cuypers *et al.*, 2013 ▸). These data were collected from a 6.9 mm^3^ perdeuterated crystal of RdPf using the monochromatic thermal neutron diffractometer D19 at the ILL. Although more commonly used for diffraction studies in structural chemistry, the D19 diffractometer is capable of collecting data to atomic (and potentially sub-atomic) resolution from small macromolecules (cell edges <50 Å) and to medium resolution (1.8–2.5 Å) from larger macromolecules (cell edges <100 Å), provided that sufficiently large crystals of several mm^3^ are available. This has been demonstrated in recent years by the ensemble of neutron studies of xylose isomerase (MW ∼43 kDa) (Kovalevsky *et al.*, 2010 ▸, 2012 ▸; Langan *et al.*, 2014 ▸).

## 4. Neutron instrumentation at spallation neutron sources   

At spallation neutron sources, neutrons produced by proton pulses are ‘time-stamped’ such that, by recording TOF information, the corresponding energy and wavelength of each neutron can be calculated. TOF techniques in combination with large position-sensitive detectors (PSDs) allow wavelength-resolved Laue patterns to be collected using all the available neutrons (Langan *et al.*, 2004 ▸). The TOF Laue method therefore has all of the advantages of quasi-Laue methods employed at reactor sources, but does not suffer in the same way from reflection overlap and a build up of background scattering over the wavelength range. In the last few years, two new TOF Laue diffractometers dedicated to macromolecular crystallography have been constructed; MaNDi (Coates *et al.*, 2010 ▸) at the Spallation Neutron Source (SNS) at ORNL and iBIX (Kusaka *et al.*, 2013 ▸) at the Japan Proton Accelerator Research Complex (J-PARC). Table 2 ▸ provides details of diffractometers currently in operation at reactor and spallation neutron sources.

## 5. Neutron cryo-crystallography   

As neutrons of the energies used for crystallographic experiments do not cause any radiation damage, data collection is generally performed at room temperature. Nevertheless, the ability to collect data at cryogenic temperatures is of interest. Since, the reduction of dynamic disorder at cryogenic temperatures lowers atomic displacement parameters (ADPs), this leads to improvements in the nuclear scattering density definition (Blakeley *et al.*, 2004 ▸) and permits the use of smaller crystals [*cf.* room temperatue (RT)] to achieve equivalent or potentially higher resolution data. In addition, certain crystals are unstable at RT and so the ability to cryo-cool crystals for data collection increases the array of feasible experiments. Moreover, comparison of structures determined at different temperatures can be made. This is important since protonation states can change due to the p*Ka* dependence on temperature. Furthermore, as the vast majority of X-ray structures are determined at 100 K, it is important to examine alterations to macromolecular structures caused by cryo-cooling, without the need to deconvolute from radiation damage effects. Finally, cryo-crystallography allows more sophisticated experiments to be performed, such as cryo-trapping of enzymatic reaction intermediates (Casadei *et al.*, 2014 ▸). Data collection at cryogenic temperatures is now possible on BioDIFF, LADI-III, D19, iBIX and MaNDi in a routine manner analogous to X-ray data collections at 100 K, *i.e.* using standard cryo pins, loops *etc.* (Coates *et al.*, 2014 ▸).

## 6. Structural refinement options for neutron diffraction data   

In the last few years, new structural refinement tools have been developed which allow structural refinement against neutron data alone, or in a joint refinement strategy with both X-ray and neutron diffraction data. With the addition of H and D atoms, a neutron structure has approximately twice as many atoms as an X-ray structure. As such, attempting to refine a neutron structure at medium resolution (*e.g.* *d*_min_ from 2.0 to 2.5 Å), particularly those with large unit cells, can be problematic as the data-to-parameter ratio is low. By combining data from both X-ray and neutron techniques, the data-to-parameter ratio is increased, while the influence of systematic errors can be reduced. Joint X-ray/neutron refinement strategies make it possible to allow refinement of all the atoms within the structure, in principle resulting in more accurate structures. The *phenix.refine* program (Afonine *et al.*, 2010 ▸), for example, is able to refine structures in a joint X-ray/neutron strategy, as well as against neutron data alone. In addition, improvements made to *SHELXL2013* (Gruene *et al.*, 2014 ▸) make the program more convenient to use for refinement of macromolecular structures against neutron data.

## 7. Recent instrumentation development for sub-atomic resolution X-ray structures   

Since the discovery of X-rays just over 100 years ago, the brilliance of X-ray sources *via* the SR sources has increased by ten orders of magnitude. It is only 35 years since the world’s first dedicated X-ray source, SRS at Daresbury, designed and built for the purpose, emerged providing nearly a billion times increase in X-ray beam brilliance compared with the best X-ray tubes at the time. The ever-increasing thirst for more and more intense X-ray beams has led to several advanced compact third-generation SR sources in the last decade, SOLEIL (2006), Diamond (2007) and the Shanghai Synchrotron Radiation Facility (2009) bringing beam brilliance similar to or exceeding {10^17^–10^20^ photons [s mm^2^ mrad^2^ (10^−3^ bandwidth)]^−1^} the large-circumference (>1 km) storage rings, 6 GeV ESRF (1993), 8 GeV SPring-8 (1997) and 7 GeV APS (1998), that came into operation before the turn of the of the century. The last decade saw many of the storage rings introduce top-up mode of operation where a steady current in the storage ring is maintained by periodically injecting small amounts of current providing a much more stable beam in both the storage ring and on the beamlines by keeping the heat loads constant over long time periods. Despite the tremendous progress in the performance of SR sources in terms of brightness during the last decades, where the brightness gain has been twice as fast as the rate of improvement in semiconductors (Moore’s law), the high horizontal emittance has remained the restricting factor in achieving the ultimate brightness of the beam. A radical reduction in horizontal emittance can be achieved by building the ring with a large number of focusing cells containing a bending magnet as well as higher-order multipole magnets necessary for focusing the electron beam. The technological development of non-evaporable getter (NEG) pumps for pumping vacuum systems originally undertaken at CERN has allowed a drastic reduction in the magnetic gaps. This is deployed in the two innovative rings MAX-IV (Sweden) and Sirius (Brazil) that are being constructed using the multi-bend achromat lattice to achieve a diffraction-limited storage ring. The horizontal emittance of these rings is expected to be 3 × 10^2^ pm rad bringing the average brightness of 10^22^ photons [s mm^2^ mrad^2^ (10^−3^ bandwidth)]^−1^ to the experiments before the end of the decade (Eriksson *et al.*, 2014 ▸). Many of the major facilities including ESRF, APS and SPring-8 are upgrading their sources using these new technological breakthroughs.

The increasing X-ray photon density delivered by the increasingly brighter sources has required rapid development in beamlines, optical elements and detectors. For X-ray macromolecular crystallography, the second-generation sources initially used an asymmetric-cut crystal to compress the beams horizontally and a mirror to compress the beam vertically. Towards the end of the last century many advances took place allowing the use of XAFS-type monochromators with the second crystal providing the horizontal focus and maintaining the fixed beam position. These also provided the full tunability across the wavelengths from ∼2.5 Å for sulfur phasing to ∼0.4 Å for ultra-high resolution at SPring-8. The last ten years has seen major progress in the provision of micro-focus dedicated facilities at several sources. The pioneering work for micro-focus beam was carried out by Riekel at ID13 at the ESRF at the start of the century (Riekel, 2004 ▸). The use of X-ray waveguide optics (Mo/C/Mo sandwich structure with a carbon spacer of 80 nm) can provide beams of 0.1 µm in one dimension (Müller *et al.*, 2000 ▸). X-rays are transported in the waveguide through the light element layer by total reflection at the opposing metallic layers. A graded multilayer mirror can be used to focus the beam horizontally to 3 µm while the waveguide can focus the beam to 0.1 µm in the vertical direction (Fischetti *et al.*, 2009 ▸). The (GM/CA-CAT) dual canted undulator beamlines at the APS that played a major role in the 2012 Nobel Prize has developed a ‘mini-beam’ apparatus that conditions the focused beam (20 µm × 65 µm) to either 5 µm or 10 µm (FWHM) diameter with high intensity. Recently a quad mini-beam collimator has been implemented which is able to deliver high intensity (5 × 10^10^ and 3 × 10^9^) with a minimum focal size of 5 and 1 µm at the sample position. There are now some 24 micro-focus macromolecular crystallography beamlines delivering beams of 5–20 µm on the sample over a broad energy X-ray range from 3.5 to 35 keV (Smith *et al.*, 2012 ▸).

**Figure 2:**
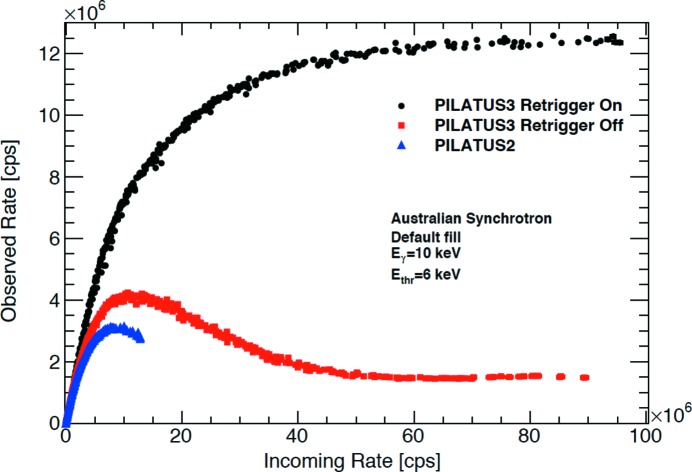
The PILATUS detectors have continued to develop and improve the spatial resolution, count rate, readout speed as well as sensitivity across the wavelength ranges. Most recently a unique in-vacuum X-ray detector, PILATUS 12M-DLS, has been installed on the I23 beamline at Diamond Light Source for long-wavelength X-ray crystallography. The PILATUS 12M-DLS is a semi-cylindrical detector covering a 2θ range of ±100° enabling the collection of low- and high-resolution data simultaneously. (Data were provided by Dr Clemens Schulze-Briese comparing PILATUS3 and PILATUS2.)

The increase in X-ray brilliance has required major improvement in the X-ray detectors. At the second-generation sources such as SRS (Daresbury), Photon Factory and NSLS the early data were collected on photographic films until the image-plate systems were introduced. The image-plate systems were initially used in off-line mode where the scanning of plates was required during data collection. The 1990s saw the development of on-line image-plate systems where reading and erasing could be performed without actually physically handling an image plate (Amemiya, 1997 ▸). The charged coupled device (CCD) detectors emerged at the end of the last century with several commercial companies providing continued improvement in the size, pixel resolution, speed of readout and sensitivity at prices that could be afforded by most SR centres for their crystallographic facilities. The last ten years have seen the development of photon-counting hybrid pixel array silicon detectors (Broennimann *et al.*, 2006 ▸) bringing shutterless data collection routine for SR crystallography. These detectors have provided very high dynamic range, zero dark signal and zero readout noise and hence are able to achieve optimal signal-to-noise ratios at short readout time and high frame rates. Fig. 2 ▸ shows the rate response for the PILATUS3 series of detectors.

## 8. Redox biology: from supply to utilization of electrons   

Many biological processes rely on redox processes that utilize the redox properties of a metal centre or cluster. Atomic- to sub-atomic-resolution X-ray structures are capable of providing structural information at an unprecedented level of detail but a major challenge remains that redox centres are the first to be affected by photoreduction (Yano *et al.*, 2006 ▸; Hough *et al.*, 2008 ▸; Ellis *et al.*, 2008 ▸; Kekilli *et al.*, 2014 ▸). Several solutions are being developed to overcome this, including deploying large crystals in combination with micro-focus beams with rapid CCD and photon-counting detectors. The use of ultra-bright femtosecond pulses from XFELs are allowing most damage processes to be outrun and have recently enabled structure determination of the fully oxidized resting state of bovine cytochrome *c* oxidase (Hirata *et al.*, 2014 ▸) and the undamaged oxygen-evolving complex of photosystem II at the SACLA FEL (Suga *et al.*, 2015 ▸), bringing the details of Mn clusters in PSII to be in complete agreement with XAFS. In order to minimize radiation damage, the crystal can be translated every few degrees of data so as to provide the structure of a pure redox state, rather than a mixture generated by X-ray radiolysis. This development can be combined with neutron diffraction studies, since the crystal can be first used to collect high-resolution neutron diffraction without any radiation damage issues, before obtaining a sub-atomic-resolution X-ray structure from the same crystal. One such example where this has been achieved is the high-potential iron–sulfur protein (HiPIP). HiPIP has a MW of ∼9 kDa and possesses an Fe_4_S_4_ cluster, which exhibits +2/+3 redox states and acts as an electron carrier from cytochrome *bc*1 complex to the reaction centre complex in photosynthetic purple bacteria. Using a D-exchanged HiPIP crystal with volume of 2.3 mm^3^, neutron data were collected to 1.17 Å resolution using the TOF Laue diffractometer iBIX at J-PARC. Subsequently, X-ray data to 0.48 Å resolution were collected using short X-ray wavelengths (0.4 Å) and an intense micro-focus beam, together with translation of the large crystal during data collection (Hirano & Miki, unpublished results; data presented at IUCr congress 2014). The X-ray resolution achieved for HiPIP equals the record held until recently by the much smaller protein, crambin (presented at the IUCr Congress 2014), which has a MW of ∼5 kDa. A joint X-ray/neutron structural refinement of these very high-resolution data sets will certainly contribute to our understanding of the coupling of electron and proton transfer dynamics.

**Figure 3:**
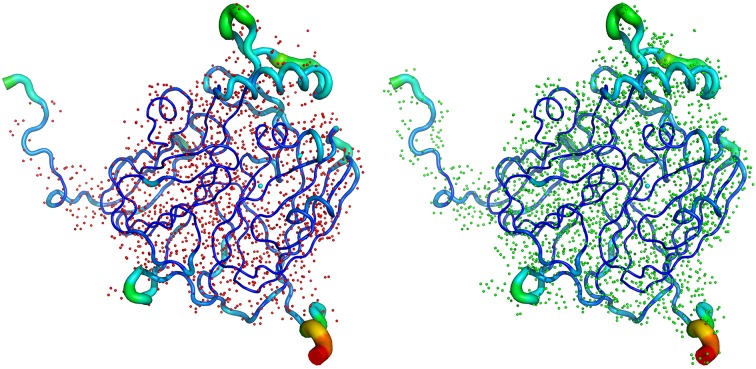
Left: the X-ray structure of *Ac*NiR (from *Achromobacter cycloclastes*), showing the number of H atoms visible (1649) in the electron density maps at 0.87 Å resolution. Right: the same X-ray structure of *Ac*NiR but showing all 2700 expected H atoms in the structure; 61% of expected H atoms are observed in this 0.87 Å resolution X-ray structure.

**Figure 4:**
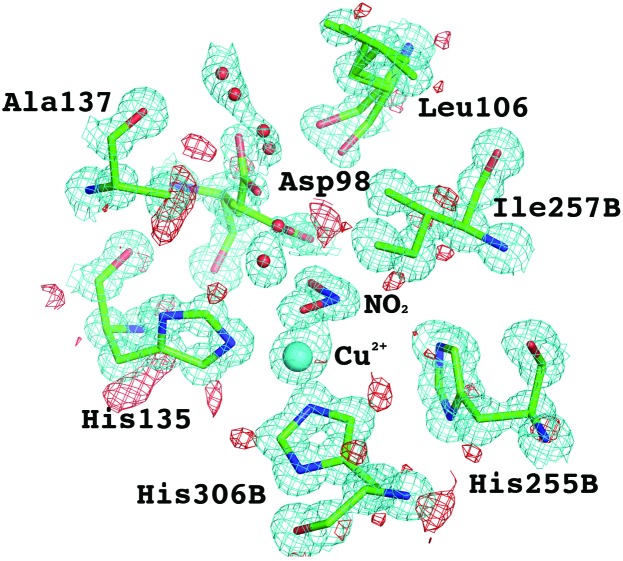
The type 2 Cu site of *Ac*NIR with nitrite bound at 0.87 Å resolution. The catalytically important residue Asp98 is seen in multiple conformation with two distinct conformations visible. The 2*F*_o_ − *F*_c_ electron density map (in cyan) is contoured at the 1.5σ level and the *F*_o_ − *F*_c_ hydrogen omit map (in red) is contoured at the 2.0σ level.

The coupling of electron and proton transfer takes place in copper nitrite reductases (NiRs) and this has been demonstrated through extensive structure–function studies of these enzymes (Antonyuk *et al.*, 2005 ▸, 2013 ▸; Leferink *et al.*, 2011 ▸). Here, we include the results of a 0.87 Å resolution X-ray structure of NiR from *Achromobacter cycloclastes* (*Ac*NiR) (MW ∼37 kDa) with its substrate nitrite bound. The structure contains all intrinsic residues (residues 4–340), two Cu ions, two malonate ions, a nitrite ion, three acetate ions, a sulfate ion and 674 water molecules. The final *R*_work_ and *R*_free_ factors were 12.3% and 13.9%, respectively. Data-collection and processing statistics for the X-ray data are given in Table 3 ▸. Due to the very high resolution of the X-ray data,^1^ 61% of expected H atoms are observed in this structure (Fig. 3 ▸). Fig. 4 ▸ shows nitrite-bound active site with a significant number of H atoms visible for the catalytic core. Nevertheless, despite the large proportion of H atoms visible, many of the key H atoms of the proton pathways are not discernible. Thus, multiple conformations observed for Asp98, representing different reaction states, make it impossible to see protons (Fig. 4 ▸) associated with this proton-abstracting residue. This has prompted us to initiate neutron crystallographic studies on *Ac*NiR for which large crystals can be grown (see below).

## 9. Neutron diffraction data collection and processing for *Ac*NiR   

**Figure 5:**
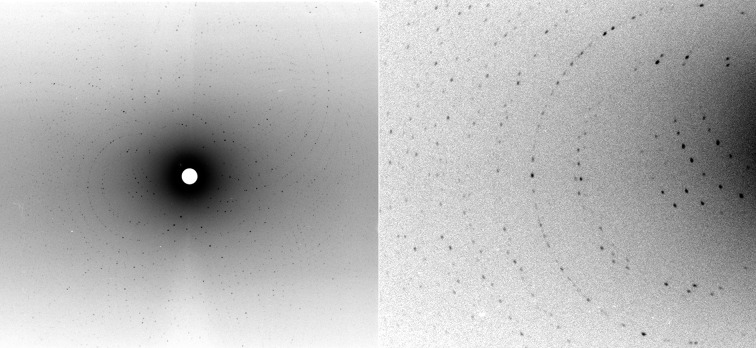
Left: a neutron quasi-Laue diffraction pattern from a D-exchanged crystal of *Ac*NiR (volume = 0.3 mm^3^) collected using the quasi-Laue LADI-III diffractometer. Neutron diffraction data were processed to 2.3 Å resolution. Right: zoomed-in close-up of part of the quasi-Laue diffraction pattern.

**Figure 6:**
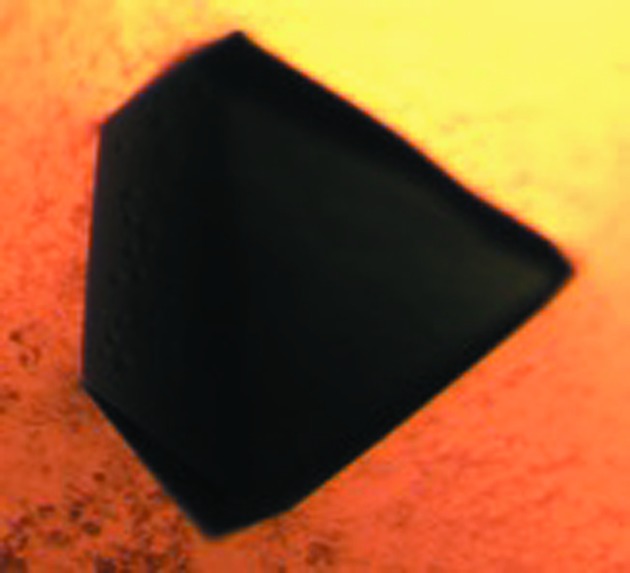
Large crystals of perdeuterated *Ac*NiR have been recently grown and one of the largest (∼1 mm^3^) is shown here.

The largest crystal available of *Ac*NiR with volume ∼0.3 mm^3^ (0.8 × 0.7 × 0.6 mm) was soaked in D_2_O buffers one month prior to neutron data collection, in order to exchange labile H atoms for D atoms. Using exposures from 12 to 24 h, diffraction data at RT were collected from the D-exchanged crystal to 2.3 Å resolution (Fig. 5 ▸) over a period of 15 days using LADI-III. Data were indexed and integrated using *LAUEGEN* (Campbell *et al.*, 1998 ▸), wavelength-normalized using *LSCALE* (Arzt *et al.*, 1999 ▸) and scaled and merged using the CCP4 program *SCALA* (Winn *et al.*, 2011 ▸). Data-collection and processing statistics for the neutron data are given in Table 4 ▸. Structural refinement using the neutron data is currently in progress and will be reported elsewhere. In addition, perdeuterated *Ac*NiR has since been produced in the Deuteration Laboratory at the ILL, and larger crystals of ∼1 mm^3^ have been grown (Fig. 6 ▸). In summer 2015, we hope to collect a complete data set using LADI-III on these larger perdeuterated *Ac*NiR crystals, with the aim to extend the resolution of the diffraction data such that the visibility of all H atoms (as D atoms) in the proton pathways will be enhanced.

## 10. Molecular sensing: heme-based gas sensors   

**Figure 7:**
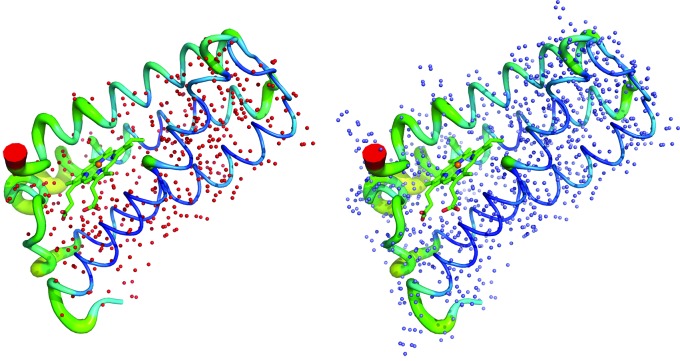
Left: the 0.84 Å resolution X-ray structure of *Ax*CytCp, showing the number of H atoms visible (694) in the *F*_o_ − *F*_c_ hydrogen omit map (in red), contoured at the 2.0σ level. Right: the same X-ray structure of *Ax*CytCp but showing all 989 expected H atoms in the structure.

**Figure 8:**
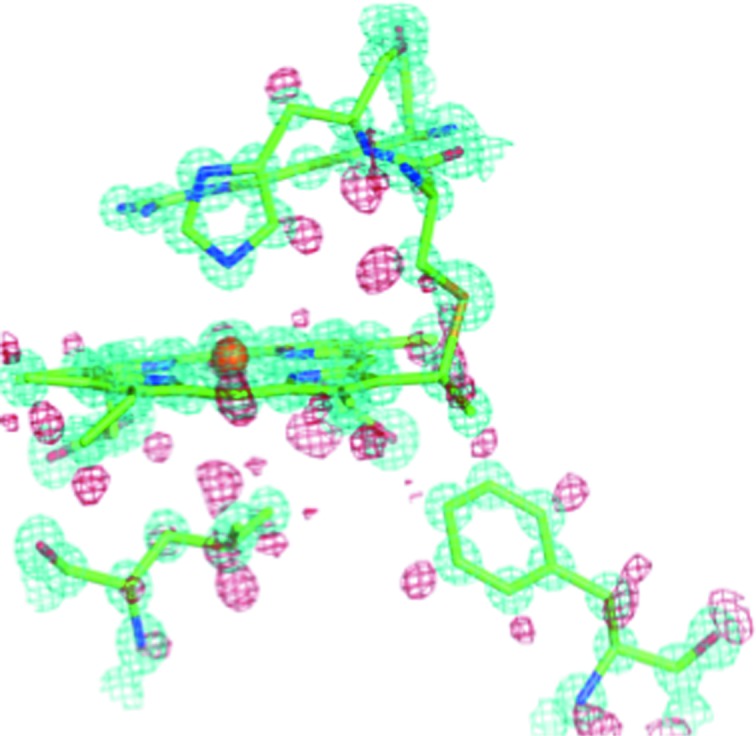
The heme binding pocket of *Ax*CytCp at 0.84 Å resolution, showing electron density for H atoms (in red) associated with key residues Leu, Phe or Met. The 2*F*_o_ − *F*_c_ electron density map (in cyan) is contoured at 1.5σ, and the *F*_o_ − *F*_c_ hydrogen omit map (in red) is contoured at 2.0σ.

In biology, substrates have distinctive sizes, shapes and charge distributions that are specifically recognized by their target biological partner proteins. The discrimination between the gases O_2_, NO and CO by heme proteins is a remarkable example of biological specificity because these molecules are apolar and of very similar size. Molecular recognition of these gases is essential for respiration, cell-signaling, aero/chemotaxis and regulation of gene expression. Cytochrome *c*′ (CytCp) belongs to a family of pentacoordinate (5c) heme proteins that discriminate between these diatomic gases efficiently helping protect bacteria from nitrosoative stress and/or NO shuttling during denitrifcation. The best characterized CytCp (MW ∼14 kDa) is from the denitrifying bacterium *Alcaligenes xylosoxidans* (*Ax*) (Hough *et al.*, 2011 ▸), which does not form a stable complex with O_2_, binds CO weakly as a distal six-coordinate (6c) heme-carbonyl (6c-CO), and reacts with NO to form a unique proximal 5c heme-nitrosyl (5c-NO) *via* a distal 6c heme-nitrosyl (6c-NO) intermediate. This utilization of both faces of the heme in ligand binding is unprecedented. The crowded distal heme pockets of all CytCp proteins contain a non-polar residue (Leu, Phe or Met) close to the Fe, which enforces selectivity in exogenous ligand binding. Here, we include results of a 0.84 Å resolution X-ray structure of CytCp from *Achromobacter xylosoxidans* (*Ax*CytCp) (PDB code 2YKZ) (Antonyuk *et al.*, 2011 ▸). Due to the very high resolution of the X-ray data, 694 H atoms are observed from the 989 expected H atoms, *i.e.* 70% of expected H atoms (Fig. 7 ▸). Fig. 8 ▸ shows the heme binding pocket with some of the H atoms visible for key residues Leu, Phe and Met, which play an important role in ligand discrimination. Nevertheless, not all of the H atoms are visible in the ligand-discriminatory pocket thus warranting neutron crystallographic studies of *Ax*CytCp.

## 11. Neutron diffraction data collection and processing for *Ax*CytCp   

The largest crystal of *Ax*CytCp with volume ∼1.5 mm^3^ (3.2 × 0.7 × 0.65 mm) was soaked in D_2_O buffers one month prior to neutron data collection in order to exchange labile H atoms for D atoms. Using exposures from 3–18 h, diffraction data at RT were collected from the D-exchanged crystal to 2.1 Å resolution over a period of ten days using LADI-III. Data were indexed and integrated using *LAUEGEN* (Campbell *et al.*, 1998 ▸), wavelength-normalized using *LSCALE* (Arzt *et al.*, 1999 ▸) and finally scaled and merged using the CCP4 program *SCALA* (Winn *et al.*, 2011 ▸). Data-collection and processing statistics for the neutron data are given in Table 5 ▸. Structural refinement using the neutron data is currently in progress and will be reported elsewhere. As the attempt to produce perdeuterated *Ax*CytCp has failed thus far, new D-exchanged crystals with improved volumes (∼2.0 mm^3^) have been grown. In July 2015, we hope to collect a complete data set using LADI-III on these larger *Ax*CytCp crystals with the aim to extend the resolution yet further and improve the precision and accuracy of the resulting structure.

## 12. Conclusions   

Due to the recent enhancements in capability and capacity for neutron macromolecular crystallography there has been a marked increase in the number of neutron structures deposited in the PDB. The limits of the field continue to expand with new examples illustrating further reductions in crystal volume or data collection time, and increases to resolution or unit-cell volume achievable. Moreover, the feasibility of neutron cryo-crystallography has been demonstrated, allowing a greater array of studies to be performed. This trend is set to continue through further improvements planned to existing instrumentation, such as the addition of new detectors for the iBIX and MaNDi instruments, and the construction of new instrumentation, such as the TOF Laue diffractometer ‘NMX’ at the European Spallation Source. It is fair to say, however, that, despite all the advances in the field, relative to X-rays, significantly larger (about ten times in linear dimension) crystals will always be required for neutron diffraction studies, particularly with the drive towards the study of ever-larger macromolecules and complexes. It is in this sense that further development of instrumentation and methods for large crystal growth are required. Creation of laboratories dedicated to the optimization of crystal volume and quality, in analogous fashion to the centralized deuteration facilities, would help increase the effectiveness of neutron macromolecular crystallography. Structural biologists have been dissuaded from pursuing neutron diffraction studies because of the demanding experimental requirements, but as more examples are published that illustrate important structural information not provided by X-rays can be delivered, *and* from more feasible crystal volumes, the application of neutron crystallography will become more common. In addition to the well known strengths of neutron crystallography, it is clear that the approach has much to contribute in obtaining damage-free structures of macromolecules at ambient temperature. The use of micro-focus X-ray beams in combination with translation of crystals to a position where no damage has propagated, and the ability to merge data efficiently and accurately from serial images from different portions of crystals, is already proving effective. Efforts in understanding radiation damage processes from femtosecond pulses of X-ray FELs are continuing (see, for example, Jönsson *et al.*, 2015 ▸; Nass *et al.*, 2015 ▸) with some early successes reported on cytochrome *c* oxidase and photosystem II. Though the pathway from protein to structure remains unpredictable for both neutron and X-ray crystallography, it has become much more reliable for the latter even for membrane proteins. Significant efforts (*e.g.* perdeuteration and crystal volume/quality optimization) are required by the neutron community and facility providers to make neutron crystallography more accessible to the wider structural biology community.

## Supplementary Material

PDB reference: 5akr

## Figures and Tables

**Table 1:** Neutron coherent scattering lengths and incoherent cross sections, and X-ray scattering lengths for the common elements of a macromolecule

		Neutron coherent	Neutron	X-ray scattering lengths (10^12^cm)	
Isotope	Atomic number	scattering length (10^12^ cm)	incoherent cross section (Barn = 10^28^m^2^)	sin = 0	(sin)/ = 0.5^1^
^1^H	1	0.374	80.27	0.28	0.02
^2^H (D)	1	0.667	2.05	0.28	0.02
^12^C	6	0.665	0.00	1.69	0.48
^14^N	7	0.937	0.50	1.97	0.53
^16^O	8	0.580	0.00	2.25	0.62
^31^P	15	0.513	0.01	4.23	1.83
^32^S	16	0.280	0.00	4.50	1.90

**Table 2:** Neutron diffractometers currently in operation at reactor and spallation neutron sources BIX-3 and BIX-4 have been offline since 2011, but will be online again towards the end of 2015 once the JRR-3M reactor restarts.

Instrument name	Neutron source and power	Detector type	Data collection method
LADI-III	ILL, 58 Megawatts	Cylindrical NIP	Quasi-Laue (/ = 30%, _range_ options; 2.63.4, 3.24.2, 3.85.0, 4.15.5)
BioDIFF	FRM-II, 20 Megawatts	Cylindrical NIP	Monochromatic ( choice from 2.45.6)
BIX-3	JRR-3M, 20 Megawatts	Cylindrical NIP	Monochromatic ( = 2.9)
BIX-4	JRR-3M, 20 Megawatts	Cylindrical NIP	Monochromatic ( = 2.6)
IMAGINE	HFIR, 84 Megawatts	Cylindrical NIP	Quasi-Laue (_range_ options, 1; 23, 2.84, 3.34.5, plus 2.83.0 with / 7%)
D19	ILL, 58 Megawatts	Area PSD	Monochromatic ( choice from 0.82.4)
PCS	LANL, 0.1 Megawatts	Time-sensitive PSD	Pulsed (20Hz) white TOF Laue (_range_ 0.67)
MaNDi	SNS, 2 Megawatts	30 Anger cameras	Pulsed (60Hz or 30Hz) white TOF Laue; adjustable range ( 2.16 at 60Hz, 4.32 at 30Hz)
iBIX	J-PARC, 1 Megawatt	30 -shifting fibre detectors	Pulsed (25Hz) white TOF Laue (_range_ = 0.77.8); adjustable range ( 4.0)

**Table 3:** X-ray data-collection and processing statistics for *Ac*NiR Values in parentheses are for the highest-resolution shell.

X-ray source, instrument	Daresbury Laboratory, station 10.1
Space group	*P*2_1_3; No.198
Unit-cell parameters	*a* = 94.9, *b* = 94.9, *c* = 94.9, = 90, = 90, = 90
Wavelength ()	0.92
Resolution ()	260.87 (0.90.87)
Unique reflections	231231
Completeness (%)	99.7 (99.7)
Multiplicity	7.2 (4.7)
*I*/(*I*)	32.0 (2.5)
*R*_sym_	6.1 (46.8)
Wilson *B* (^2^)	6.3
*R*_aniso_ (%)	12.3
*R*_free_aniso_ (%)	13.9
Number of protein atoms	3639
Multiple occupancy side/main chains	42/15
Water molecules	674
Partial waters	94
Cu ions	2
Sulfate ions	0.4
Acetate ions	3
Malonate ions	2
Visible/expected H atoms	1649/2700
PDB access number	5akr

*R*_sym_ = |I *I*|/*I*, where *I* is the observed intensity and *I* is the average intensity of multiple symmetry-related observations of that reflection. *R*_cryst_ = ||
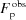
| |
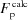
||/|
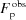
|. *R*_free_ = ||
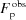
| |
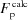
||/|
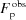
| where |
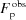
| is from a test set not used in the structural refinement. *R*_aniso_ and *R*_free_aniso_ are as *R*_cryst_ and *R*_free_, except with anisotropic atomic displacement parameter refinement.

**Table 4:** Neutron data-collection and processing statistics for *Ac*NiR Values in parentheses are for the highest-resolution shell.

Neutron source, instrument	ILL, LADI-III	
Space group	*P*2_1_3; No. 198	
Unit-cell parameters	*a* = 96.3, *b* = 96.3, *c* = 96.3, = 90, = 90, = 90	
Wavelength range ()	3.134.05	
Number of images	16	
Angle between images ()	7	
Average exposure time (min)	1365	
Resolution ()	402.3 (2.422.3)	
Total number of reflections	61260 (4095)	
Unique reflections	10414 (1273)	
Completeness (%)	78.2 (66.0)	
Multiplicity	5.9 (3.2)	
*I*/(*I*)	8.6 (5.7)	
*R*_merge_	0.182 (0.219)	
*R*_p.i.m_	0.062 (0.112)	

**Table 5:** Neutron data-collection and processing statistics for *AxCytCp* Values in parentheses are for the highest-resolution shell.

Neutron source, instrument	ILL, LADI-III
Space group	*P*6_5_22; No. 179
Unit-cell parameters	*a* = 53.0, *b* = 53.0, *c* = 177, = 90, = 90, = 120
Wavelength range ()	3.104.05
Number of images	16
Angle between images ()	7
Average exposure time (min)	934
Resolution ()	44.412.1 (2.212.1)
Total number of reflections	51767 (3500)
Unique reflections	6316 (682)
Completeness (%)	70.9 (54.4)
Multiplicity	8.2 (5.1)
*I*/(*I*)	11.2 (6.5)
*R*_merge_	0.143 (0.216)
*R*_p.i.m_	0.042 (0.081)

## Footnotes

1 Data were collected from a (0.3 × 0.3 × 0.3 mm) *Ac*NiR crystal using X-rays of 0.92 Å wavelength at 100 K on a MAR225 CCD detector on station 10.1 at the second-generation SR source, Daresbury Laboratory (Ellis *et al.*, 2008 ▸). Data extending to 0.87 Å resolution were integrated and scaled by *HKL2000*, with initial refinement performed by *REFMAC* with the final stages of refinement undertaken by *SHELX* (Sheldrick, 2008 ▸). Partially occupied waters and residues with multiple conformations were built in manually after inspection of 2*F*_o_ − *F*_c_ and *F*_o_ − *F*_c_ electron density maps. Only H atoms visible in *F*_o_ − *F*_c_ difference maps at ≥2σ were included in the final model.
